# Foraging decisions underlying restricted space use: effects of fire and forage maturation on large herbivore nutrient uptake

**DOI:** 10.1002/ece3.2304

**Published:** 2016-07-23

**Authors:** Edward J. Raynor, Anthony Joern, Jesse B. Nippert, John M. Briggs

**Affiliations:** ^1^ Division of BiologyKansas State UniversityManhattanKansas66506; ^2^ Present address: School of Natural ResourcesUniversity of Nebraska‐LincolnLincolnNebraska68583‐0961

**Keywords:** Ecosystem interactions, fire, grazing, Konza Prairie Biological Station, mesic tallgrass prairie, patch selection, stable isotope

## Abstract

Recent models suggest that herbivores optimize nutrient intake by selecting patches of low to intermediate vegetation biomass. We assessed the application of this hypothesis to plains bison (*Bison bison*) in an experimental grassland managed with fire by estimating daily rates of nutrient intake in relation to grass biomass and by measuring patch selection in experimental watersheds in which grass biomass was manipulated by prescribed burning. Digestible crude protein content of grass declined linearly with increasing biomass, and the mean digestible protein content relative to grass biomass was greater in burned watersheds than watersheds not burned that spring (intercept; *F*_1,251_ = 50.57, *P* < 0.0001). Linking these values to published functional response parameters, *ad libitum* protein intake, and protein expenditure parameters, Fryxell's (*Am. Nat*., 1991, **138**, 478) model predicted that the daily rate of protein intake should be highest when bison feed in grasslands with 400–600 kg/ha. In burned grassland sites, where bison spend most of their time, availability of grass biomass ranged between 40 and 3650 kg/ha, bison selected foraging areas of roughly 690 kg/ha, close to the value for protein intake maximization predicted by the model. The seasonal net protein intake predicted for large grazers in this study suggest feeding in burned grassland can be more beneficial for nutrient uptake relative to unburned grassland as long as grass regrowth is possible. Foraging site selection for grass patches of low to intermediate biomass help explain patterns of uniform space use reported previously for large grazers in fire‐prone systems.

## Introduction

For large grazers, the spatial distribution of forage and its associated nutritive value are fundamental components that underlie foraging behavior, resource selection, and space use (Bailey et al. 1996; Prins and van Langevelde 2008). Identifying the determinants of the distribution of grazing herbivores is a major issue facing animal and rangeland managers, and understanding how land management can guide fine‐scale, foraging decisions that drive animal distribution is paramount to wildlife management and conservation. Accordingly, optimal foraging theory assumes that foraging decisions by herbivores should be strongly influenced by physiological and environmental constraints on rates of energy and nutrient uptake. Two constraints frequently invoked for large, vertebrate grazers are the effect of plant density on the short‐term rate of food intake (availability constraint) and the effect of digestive capacity on the long‐term rate of energy/nutrient assimilation (processing constraint) (Belovsky 1978; Owen‐Smith and Novellie 1982; Belovsky 1986).

The short‐term intake rate of food (i.e., the functional response) is known to be positively correlated with plant size, bite size, and plant density (Spalinger and Hobbs 1992), yet an asymptote in intake rate is reached at high levels of plant density or biomass. The digestive capacity of herbivores is primarily governed by the interaction of energy/nutrients and fiber in their diet. As fiber content in stems increases in maturing vegetation, forage nutritive value drops and digestibility is reduced; thus, the processing constraint is affected strongly by forage quality in ruminants (Belovsky 1978; McNaughton 1985; Hobbs 1990; Van Soest 1994). The processing time (digestion and passage) in the gut often increases as plants mature (Blaxter et al. 1961; White 1983), which suggests that both digestibility and the rate of turnover of ingesta should be negatively related to plant biomass, if biomass is positively associated with plant maturation stage.

**Figure 1:**
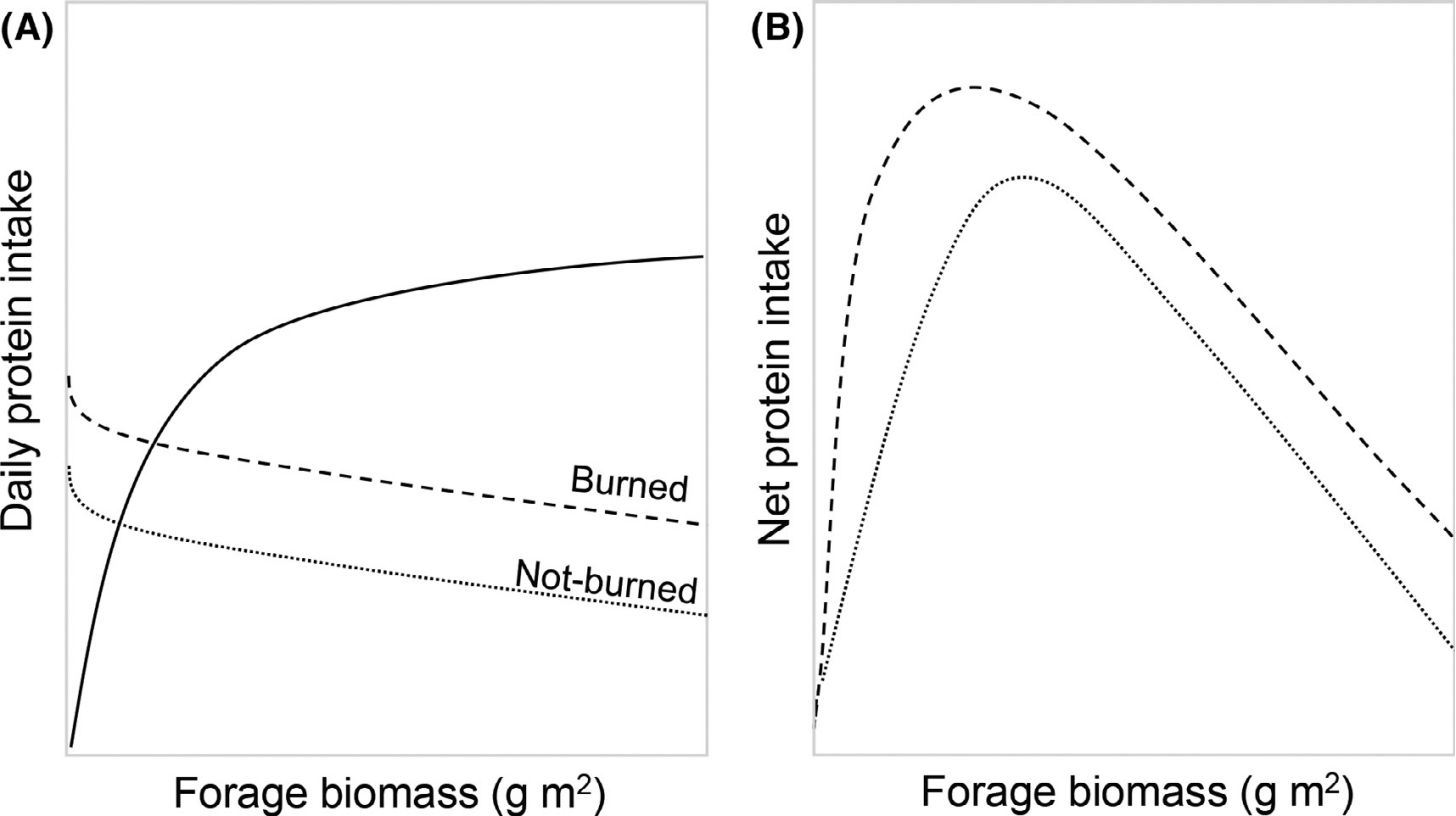
Schematic showing general mechanism of the forage maturation hypothesis (FMH) and its extension to fire‐prone ecosystems. (A) Foraging constraints of daily cropping (solid line) and digestion (dashed for burned grassland, dotted line for not‐burned grassland) that results in (B) maximum daily nutrient intake at an intermediate forage biomass in recently burned and not recently burned grassland. This figure is modified from Hebblewhite et al. (2008) for a fire‐prone grassland with the dashed line indicating constraints in burned grassland and dotted line indicating constraints in not‐burned grassland in the second panel (B).

This inverse correlation between availability and processing constraints creates the so‐called trade‐off for grazing herbivores (McNaughton 1979; Fryxell 1991). In forage patches of low biomass, the processing rate is high but the short‐term rate of intake is low, whereas in patches of high forage biomass, the processing rate is low but the short‐term intake rate is high. The net rate of energy/protein intake for grazing herbivores can be maximized accordingly on patches of intermediate plant biomass (Wilmshurst and Fryxell 1995). This is the basis of the forage maturation hypothesis (hereafter, FMH; Fig. 1) (Fryxell 1991), which posits that foragers achieve the most energetic/nutritional gain by feeding at sites where biomass is at low to moderate levels (Hobbs and Swift 1985).

The FMH has been tested for grazing herbivores in experimental and natural grassland systems. Using elk (*Cervus canadensis*) feeding in a mosaic of grass patches that were manipulated experimentally, preference for patches of low to intermediate forage biomass closely matched the value predicted by Fryxell's (1991) model for daily rate of energy/protein intake (Wilmshurst and Fryxell 1995). Further, a test of the FMH with migratory elk in the Canadian Rocky Mountains found that migration was guided by access to intermediate forage quantity of high nutritive content (Hebblewhite et al. 2008). In a subtropical system, cattle in a South African savanna also maximized daily nutrient intake in patches of intermediate forage quantity (Drescher et al. 2006). Although the FMH helps explain foraging decisions aimed at balancing availability and processing constraints of large herbivores in grasslands, how can this prominent ecological hypothesis be extended to understand the role of land management decisions such as prescribed burning that affect grassland animal distributions?

Grass‐dominated systems, namely grasslands and savannas, account for approximately 40% of the Earth's land cover (Loveland et al. 2000). The distribution of these ecosystems is known to be controlled by a variety of factors such as climate, topography, and soil nutrients, one of the most important of which is fire (Bond and Keeley 2005; Breman et al. 2012). Fire maintains structure and function of fire‐prone communities as well as creates an “ecological magnet” for many vertebrate herbivores (Archibald et al. 2005; Klop et al. 2007; Fuhlendorf et al. 2009), resulting in heavy selection and sustained use of regrowth in postburned areas (Coppedge and Shaw 1998; Sensenig et al. 2010; Eby et al. 2014).

Fire increases leaf nutrient concentrations in postfire growth (Blair 1997) while simultaneously removing older, nonpalatable tissues making green foliar tissue more accessible (Hobbs et al. 1991; Pfeiffer and Hartnett 1995). Moreover, a postfire “pulsed” increase in forage biomass production and foliar protein content occurs in grassland when released from a period of fire suppression (Seastedt and Knapp 1993; Blair 1997). The pulse in forage resources has been linked to the selection of these burned areas by large grazers (Allred et al. 2011; Augustine and Derner 2014) yet the mechanistic understanding of the foraging currency that drives the attraction to postfire regrowth has received little attention.

An inverse relationship between leaf development stage and protein content and forage digestibility (Miller et al. 1965) predicts a positive feedback between grazing activity and forage palatability (Archibald et al. 2005), a tenet of FMH. Furthermore, forage quantity influences grazer foraging behavior by potentially impeding access to young grass tissue at the feeding station scale (Ruyle et al. 1987; Drescher et al. 2006), which can be ameliorated through fire by the removal of senesced plant material.

Greater foliar protein content in postfire regrowth than unburned grassland is believed to be a primary factor controlling this “ecological magnet” effect. However, the pulse of elevated foliar protein content relative to unburned grassland is usually short‐lived; lasting 2–5 weeks (Hobbs et al. 1991), yet large herbivores continue to select burned over unburned grassland from months to years' postignition (Schuler et al. 2006; Ranglack and du Toit 2015). The potential benefits of intense grazer space use in burned grassland beyond the pulse in foliar nutrients remain unexplored.

We tested the FMH and extended the hypothesis to understand the mechanisms behind strong selection by large herbivores for burned grassland using plains bison (*Bison bison*) feeding in an enclosed mosaic of watersheds that were manipulated experimentally with prescribed fire. We hypothesized that bison prefer to feed in recently burned prairie to maximize daily protein intake (Fig. 1A), which results in net protein intake being higher in recently burned prairie than in not recently burned prairie (Fig. 1B). Bison are large ruminants of western North America. Although bison can feed on a wide variety of plants, they are primarily grazers (Coppedge et al. 1998; Towne et al. 2005). To quantify the processing constraint in burned and not‐burned watersheds, we measured changes in digestible crude protein content in relation to grass biomass from field data. To quantify the availability constraint, we used published functional response parameter values to changes in availability of grass biomass in tallgrass prairie. We used Fryxell's (1991) net energy/nutrient intake model and data on protein utilization of foraging cattle to quantify maximization of net protein intake, the foraging currency, in relation to grass biomass in burned and unburned grassland.

To evaluate outcomes of our net protein intake model, we tested whether bison in burned and unburned watersheds selected fine‐scale, foraging sites of low to intermediate biomass over the growing season. Observations were made from spring through fall to capture seasonal variation in foraging behavior. Because availability of an important food item, warm‐season grasses, is reduced as the growing season ends in grazed, tallgrass prairie (Vinton et al. 1993; Coppedge and Shaw 1998), we assessed how sward structure of feeding sites and diet composition changes over the annual cycle to understand how bison respond to forage availability and how this relates to our test of the FMH in a fire‐prone system.

## Methods

### Study area

**Plate 1:**
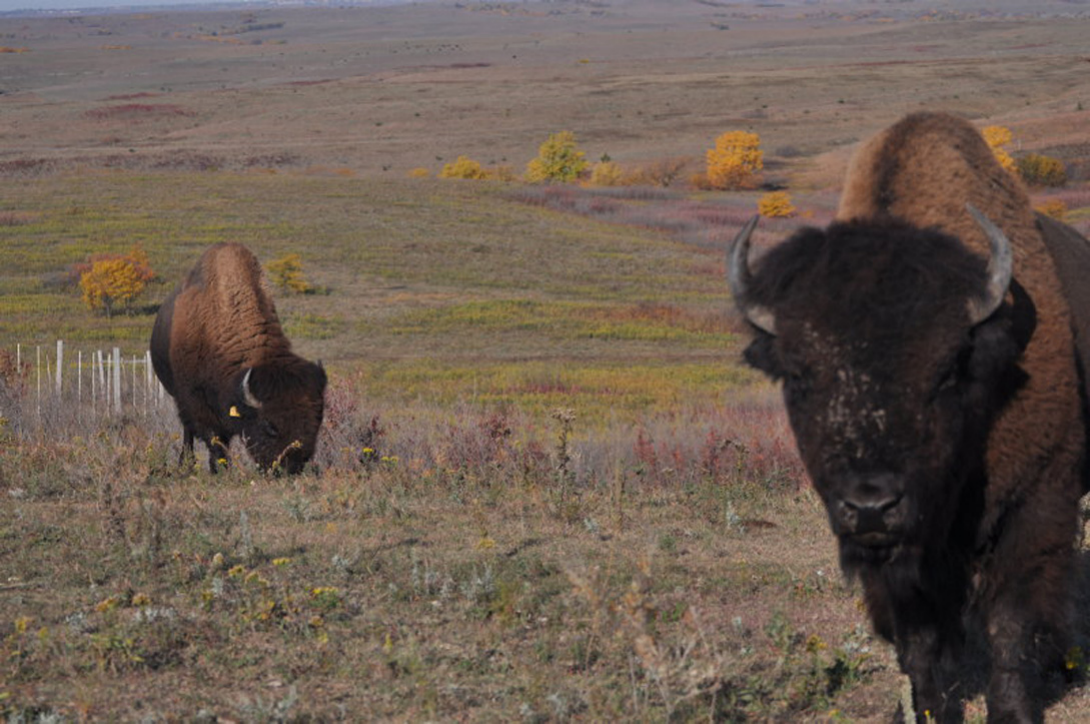
Plains bison in Konza Prairie Biological Station, Manhattan, Kansas, United States. Photograph by Edward J. Raynor.

We conducted this study in the growing season of 2012 (April to September) and summer‐fall 2013 (May to December) in a 1040 ha bison enclosure at Konza Prairie Biological Station (KPBS), near Manhattan, KS (USA) (Plate 1). KPBS is a mesic tallgrass prairie landscape that experiences a variable, continental climate with warm; wet springs; hot summers; and dry, cold winters. Mean annual precipitation is 826 mm with ~75% falling during the April–September growing season. The bison area at KPBS is managed using controlled spring burning in a replicated, watershed level experimental design, where watersheds are burned at varying frequencies (1, 2, 4, and 20 years). In general, each spring four watersheds are burned and six are not. The ten watersheds in the bison enclosure averaged 97 ha each (57–135 ha) in area. The current bison herd was established in 1987 and is currently maintained at a stocking rate of ~260 adult individuals, with ~90 calves born in each spring. This stocking rate results in a target density that removes ~25% of the standing vegetation biomass annually, on average (Knapp et al. 1999). Bison are distributed throughout the enclosure in the dormant season (December–March) with most GPS locations of ~10 GPS‐collared females in the watersheds not burned the previous spring, whereas locations switch to burned watersheds after the spring burn and remain in this habitat for the majority of the growing season (May to October) (Raynor 2015).

#### Simulating optimal nutritional intake in patches of variable biomass

We modeled the trade‐off between availability and digestion constraints following methodology proposed by Wilmshurst et al. (1995). We calculated the potential daily rate of net protein intake for bison in burned watersheds and watersheds that did not burn the previous spring for three seasons. Foraging kinetics data from bison and cattle literature were used to parameterize the model.

The nutritional intake model includes the following. The *availability constraint* is modeled as the product of the functional response and forage digestible protein (DP) content, where the functional response is an increasing function of grass biomass (Gross et al. 1993; Bergman et al. 2000; Fortin et al. 2002). The *processing constraint* is modeled as *ad libitum* intake multiplied by forage DP content. Given that passage rate limits intake (Arnold 1985) and passage is slow for poor‐quality forage (Van Soest 1994), the digestive constraint should decrease with increasing grass biomass. We assume an average body mass of 700 kg for adult bison and maximum foraging time of 10.7 h (Hudson and Frank 1987).

To estimate forage digestible crude protein (CP) content (*Q*, in g CP/kg), we used digestible CP content (DCP) and graminoid weight from clippings of pooled graminoids in foraging areas identified in foraging site selection observations described below. DCP (%) is the minimum of crude protein and indigestible crude protein from the forage sample collected in foraging areas (Goering et al. 1972). Next, we created linear regression equations for each of the three seasons and two burn types (burned or not‐burned during spring) to estimate digestible protein available at different levels of forage biomass (Table S1). This value was then multiplied by the functional response from Fortin et al. (2002) for dry matter intake of plains bison to estimate the availability constraint over the growing season and dormant season. We therefore calculated the availability constraint (*I* ^1^, in g CP/day) as

(1) $$I^{1}=\frac{QTaV}{b+V}$$

where *a* is the maximum rate of forage consumption (in kg/h), *V* is forage biomass (in kg/ha), and *b* is the biomass at which the intake rate is one‐half maximum (in kg/ha). *T* is maximum foraging time of 10.7 h (Hudson and Frank 1987).

We defined the processing constraint (*I* ^2^, in g/day) as the maximum amount of protein that a bison could digest per day given *ad libitum* consumption. To estimate the processing constraint, we multiplied the positive DCP function by an *ad libitum* intake value of ~16.5 kg/day (Holechek 1988). Foraging costs (*X*) were based on cattle (*Bos taurus*) nitrogen utilization data (0.04 g/kg body mass^0.75^) (Kohn et al. 2005) scaled to bison body size (700 kg). Net daily protein intake was estimated as *I *− *X*, where *I* is the minimum of *I* ^1^ and *I* ^2^. Finally, the daily rate of protein intake was calculated over a range of forage biomass levels (0–3300 kg/ha) measured at KPBS (Knapp et al. 1998). The peak of this relationship predicts the maximal protein intake over a range of available grass biomass.

#### Feeding‐site selection observations

To evaluate feeding‐site selection, we measured vegetation biomass (g m^−2^) and graminoid foliar protein (% CP) at active feeding sites and compared these with measurements from nearby sites located randomly within the same watershed. A feeding site was defined as an approximately circular area where >50% of the bison in a group (at least two individuals) were observed feeding together for a minimum of 30 min. Observations were taken throughout the entire daylight period. Vegetation at three feeding sites was sampled using three 1 m^−2^ plots, placed randomly within a 25 m^−2^ radius circle adjacent to the feeding‐site location; we did not sample vegetation actually eaten (Schaefer and Messier 1995). In every plot, the percent cover of graminoids, forbs, and litter was estimated visually for each vegetation group in 10% classes by the sole observer (EJR). The same sampling design was repeated at three sites not grazed during feeding observations located in a random direction from each feeding site; samples were collected at a distance of 50–75 m for comparison with feeding sites.

To estimate plant biomass, we clipped individual plants 2 cm above the ground [in 0.1 m^−2^ plots] in each of the three plots next to each feeding site and in nearby, random locations. Plant samples were dried in the field in paper bags, oven‐dried for 48 h at 60°C, and weighed. Because graminoids comprise >90% of the diet of bison in tallgrass prairie (Coppedge et al. 1998), all graminoids were separated from clipped vegetation samples, bulked, and analyzed for nutrient content. For each paired set of samples, two of the three clipped graminoid samples were randomly selected for nutrient analysis.

#### Nutrient analyses

Samples were dried, ground to a 1 mm particle size in a ball mill, and analyzed by Dairyland Laboratories Inc. (Arcadia, WI) on a Foss model 5000 NIR instrument. Graminoid crude protein and digestible crude protein contents were determined using NIRS (near‐infrared spectroscopy) analysis (calibration *R* ^2^ = 0.985). Crude protein in samples was estimated as “%‐N in plant tissue × 6.25” (Jones 1941). To gauge shifts in feeding‐site selection patterns as overall plant quality declined over the growing season, observations were combined into three seasonal periods: growing (15 April–30 June), peak mature growth (1 July–14 October), and dormant (15 October–31 December) representing periods of actively growing plants, peak mature growth, and senesced plant tissue.

### Data analyses

#### Feeding‐site selection

To test whether selected sites contained low to moderate biomass of high quality as generally predicted by the FMH, we compared graminoid biomass and graminoid crude protein content at sites selected for grazing and sites not selected for grazing using analysis of variance. Graminoid biomass at used and available foraging sites were pooled to assess consistency of the net nutrient intake model predictions for foraging areas. These values were then adjusted to kilogram per hectare. To determine whether foraging patterns varied with the progression of the growing season, an analysis of covariance was used to determine whether mean availability of green foliar tissue and mean cover of grass, forbs, and litter relative to days since May 1, the approximate end of the burning season at KPBS, was different between used and available foraging sites.

We compared feeding sites to paired random sites using conditional (i.e., case‐controlled) logistic regressions, using the *clogit* function in the library “survival” with our paired used and available sites as the strata term (R Development Core Team 2012). We included the following covariates to explain the response variable, whether the site was used (coded as 1) or not (coded as 0): (1) percentage of green foliage, (2) grasses, (3) litter, and (4) forbs. Candidate models were built to include the following: a full model, a null model (without covariate), a model for every single covariate, a set of models comprising multiple combinations of two covariates, and a set of models where percentage of green foliage were in interaction with each of the vegetation group covariates. Models were ranked based on Akaike's information criterion corrected for small sample size (AIC_c_). For each candidate model *i*, we calculated the difference between the AIC_c_ of model *i* and the AIC_c_ of the best model (AIC_c_) and Akaike weight (*ω*_i_). Models with ΔAICc < 2 were considered to have substantial empirical support, ΔAIC_c_ 4–7 considerably less, and ΔAICc > 10 essentially no support (Burnham & Anderson 2002).

#### Seasonal diet shifts using *δ* ^13^C

To assess changes in diet composition over the annual cycle, tail hair was collected from each of the same four, uniquely identified matriarchal females throughout 2010–2013 during the annual bison round‐up activities in late October of each year. In the laboratory, hair samples were cleaned with acetone to remove dirt, grit, and oils. Hairs were serially sampled in 5‐mm sections from the follicle (most recently produced) to hair tip [oldest section; similar to the procedure outlined in Cerling et al. (2009)]. We classify diets of primarily C_4_ plant as those represented by hair follicle *δ* ^13^C values greater than −14‰, while values smaller than −14‰ represented mixed C_4_–C_3_ plant diets unless a value of −25‰ was reached (Dawson et al. 2002). Supplemental material summarizes the methodology for the estimation of annual cycle diet composition.

## Results

### FMH model

**Figure 2:**
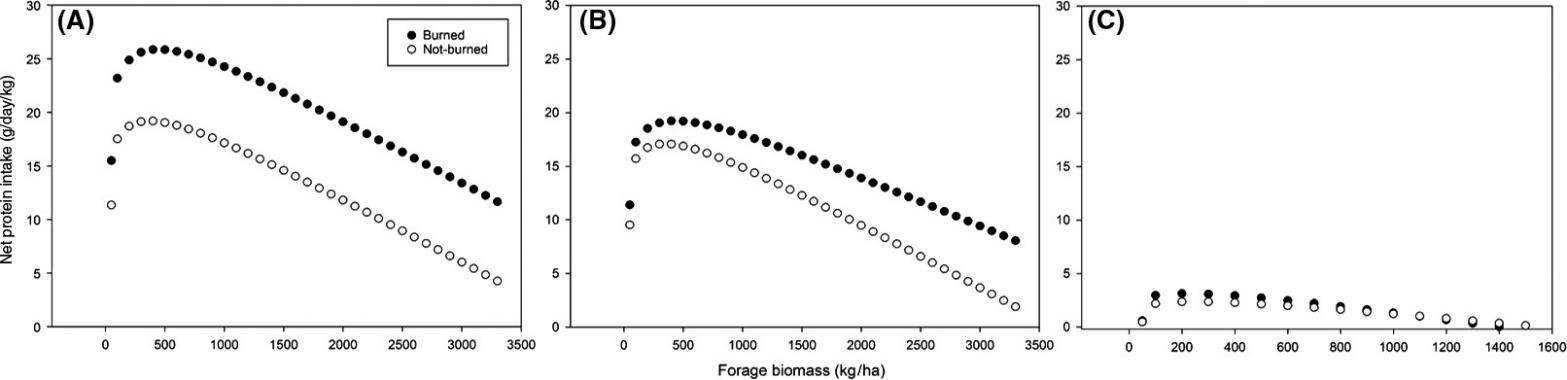
Function predicting daily net protein intake for plains bison over a range of vegetation densities in burned and not‐burned tallgrass prairie for (A) early growing season, (B) peak growth season, and (C) dormant season.

Field data pooled over the two seasons suggested graminoid DCP declined linearly with increasing biomass (kg/ha) (*y* = 5.03−0.02*x*,* F*_1,253_ = 55.55, *P* < 0.0001, *R* ^2^ = 0.18). We calculated the relationship between DCP and increasing grass biomass for each season and watershed burn status for use as seasonal watershed DCP in our net protein intake model (Table S1). By linking seasonal functional responses, watershed DCP, and grass biomass availability with estimates of nutrient expenditure in the model, we estimated daily rates of net protein intake in relation to grass biomass available at KPBS (Fig. 2). Our model predicted that daily protein intake should rise steeply at low forage biomass, peak abruptly, then slope downward with further increases in grass biomass. Peaks in daily protein intake at roughly 500, 500, and 200 kg/ha should occur in the growing, peak growth, and dormant season, respectively. At the optimal grass biomass, the maximum daily intake of protein should be 26, 19, and 3 g/kg body mass in the growing, peak growth, and dormant seasons for 700 kg bison grazing in burned tallgrass prairie. The maximum daily protein intake should be 19, 17, and 2 g/kg body mass in the growing, peak growth, and dormant seasons for 700 kg bison grazing in not‐burned sites. The difference in protein uptake between grazing at optimal biomass levels in burned and not‐burned watersheds was 27, 11, and 0.3% across the three seasons.

#### Patch use

In the bison enclosure, grass biomass within burned watersheds remained lower at foraging areas than at foraging areas in not‐burned watersheds over the growing season, ranging between an average of 790.2 kg/ha in foraging areas during the early growing season postburn and 590.6 kg/ha in foraging areas during the peak growth season. In watersheds not burned in spring, grass biomass in foraging areas ranged between 1180.8 kg/ha during the early growing season and 920.9 kg/ha in the peak growth season. These results indicate repeated grazing at foraging areas from the early growing season to the peak growth season maintained forage biomass in a state of low to intermediate availability throughout the growing season. During the dormant season, grass biomass at foraging areas in burned watersheds offered 860.2 kg/ha, while 1200.3 kg/ha was available in watersheds that did not burn that spring.

**Figure 3:**
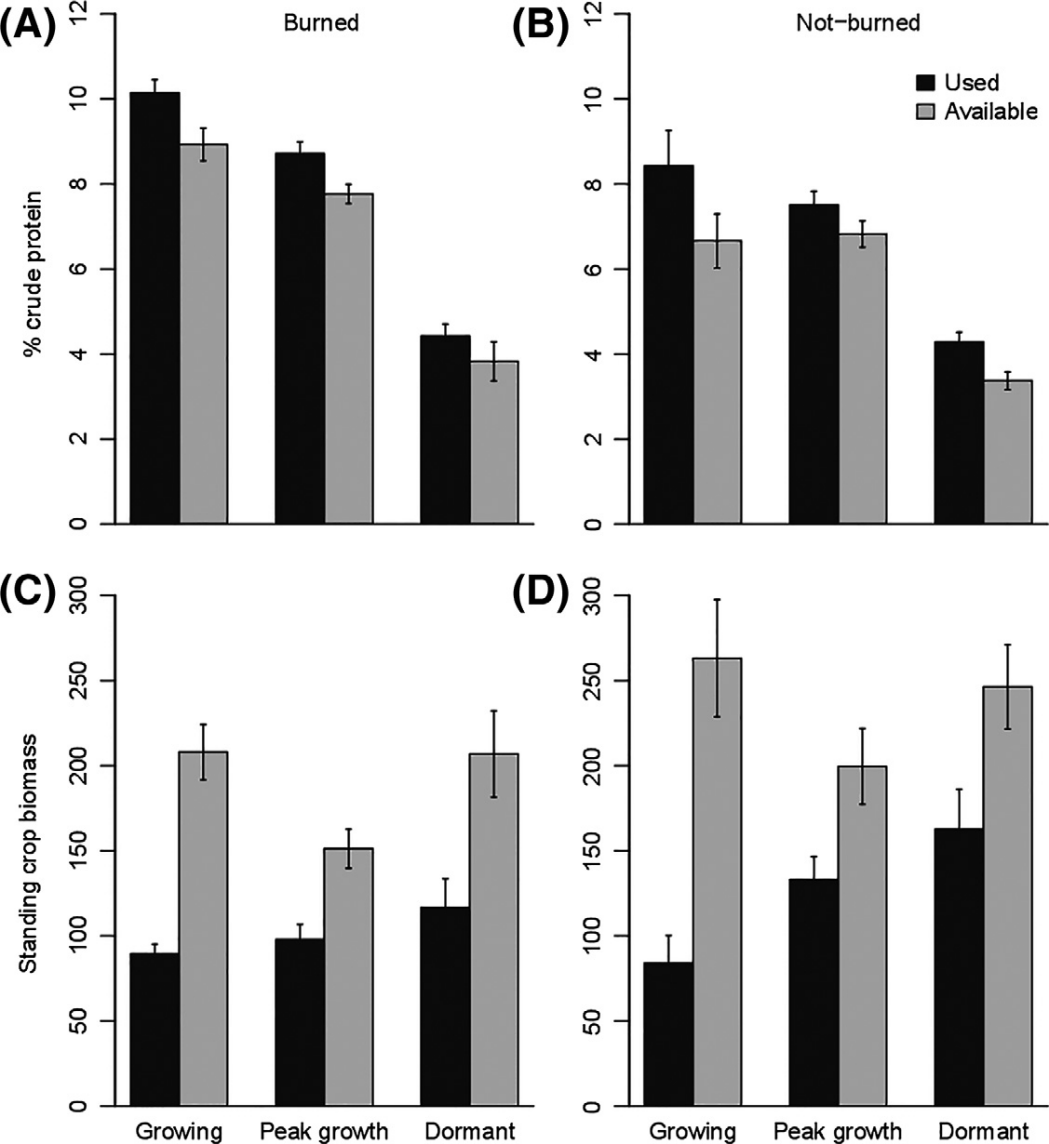
Graminoid characteristics over three seasonal periods at used, feeding sites and at nearby ungrazed but available sites in watersheds that were burned that year and watersheds that did not burn that year: (A, B) mean grass biomass (±SE) and (C, D) mean crude protein content (±SE) of graminoids. Data were collected from 2012 to 2013 and lumped in these comparisons. All feeding sites to available site comparison are significantly different (*α *= 0.05).

Overall, grass biomass in foraging areas was higher in the dormant season than during the early growing and peak growth seasons (*F*_2,129_ = 6.17, *P *=* *0.002). Foraging area grass biomass was also lower in burned watersheds than not‐burned watersheds (*F*_1,133_ = 14.86, *P *=* *0.0002), likely due to recursive grazing. Grass biomass at feeding sites was lower than that observed at nearby random locations: growing (66% lower), peak growth (41%), and dormant season (39%; *F*_1,133_ = 53.15, *P *<* *0.0001, Fig. 3A,B).

Higher crude protein content was found in graminoids during the growing season than was observed for either the peak growth or dormant season (*F*_2,128_ = 98.77, *P < *0.0001). Additionally, graminoid crude protein content was greater in burned watersheds across all seasons: growing (27% higher), peak growth (14%), and dormant season (8%; *F*_1,123_ = 39.29, *P* < 0.0001). Across seasons, graminoid crude protein at sites selected for grazing was higher than protein content at sites not selected for grazing during foraging observations (*F*_1,121_ = 71.51, *P *<* *0.0001, Fig. 3C,D).

The availability of green foliar tissue decreased with increasing days since May 1 (simple linear regression; *F*_1,253_ = 463.1, *P* < 0.0001, *R* ^2^ = 0.65). The mean green foliar tissue relative to days since May 1 did not differ significantly among used and available sites (*F*_3,251_ = 15.6, *P* = 0.43) or among burn status (*F*_3,156_ = 155.8, *P* = 0.67). Grass and litter cover at used and available foraging areas did not vary with increasing days since May 1 (*P* > 0.05). Mean forb cover at used sites increased at a greater rate than available sites as the growing season progressed (ANCOVA; *F*_3,262_ = 17.59, *P* = 0.0006, *R* ^2^ = 0.17); thus, sward structure between used and available foraging areas varied over the growing season. The percentages of forb and grass cover were the main variables associated with the selection of feeding sites during the early growing season as they were the only variables common to the models with ΔAIC_c_ < 2 (estimate for forb and grass cover from top model: ± (95% CI): −38.10 (−69.21, −6.99) and −10.34 (−18.11, −2.57), *N* = 40 paired samples; Table S2). During the peak growth season, forb and grass cover were the main variables that explained the selection of feeding sites (estimates for forb and grass cover: −21.39 (−30.90, −11.88) and −8.52 (−13.89, −3.14), *N* = 66 paired samples; Table S3). Reduced strength in avoidance of forb cover between the early growing and peak growth season (44% difference) suggests bison are more likely to use areas containing high forb cover during the peak season with mature vegetation growth. All confidence intervals overlapped 0 for the dormant season feeding‐site selection models with Δ_*i*_ < 4, suggesting little to no support for selection for one type of sward over another (*N* = 27 paired samples; Table S4).

**Figure 4:**
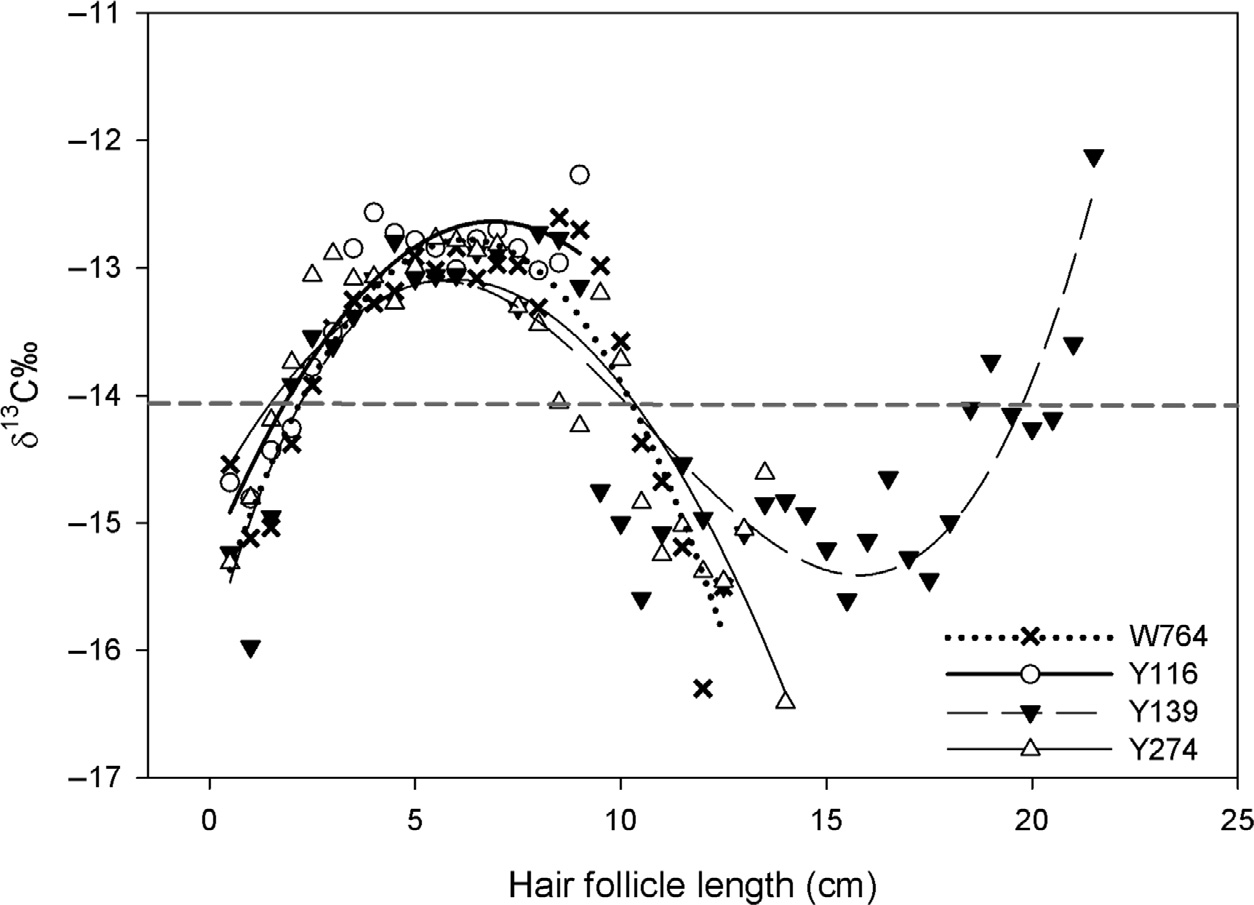
*δ* ^13^C (‰) of yearly composite hair profiles in relation to hair follicle length (cm) for four matriarchal female bison. Values represent averages of *δ* ^13^C every 5 mm over a 4‐year period (2010–2013) per individual. Hair follicle length represents the distance from the base of the follicle (collection in late October each year) to older portions of the hair closest to the hair tip. Animal Y139 had hairs of sufficient length (>20 cm) to capture an entire annual diet *δ* ^13^C profile. The horizontal dashed line indicates separation of mostly C_4_ plant diet (above line) and mixed C_3_–C_4_ diet (below line).

Segmented analysis of *δ* ^13^C in tail hairs indicated that the bison diet shifted from a strictly C_4_ plant diet during the postburn and peak mature growth seasons to a mixed diet of C_3_ and C_4_ plants in the dormant season (Fig. 4). For three of four bison with tail hairs ranging from 6 to 14 cm in length, the relationship between *δ* ^13^C signature and hair follicle length was best fit by a second‐order polynomial regression for 8 of the 12 tail hair samples. Bison #Y139 had the longest tail hairs (up to 21.5 cm) that were best fit by third‐order polynomial regression in 2010 and 2012 and fourth‐order polynomial regression in 2011 and 2013. Using the rate of tail hair growth in cattle as a growth standard, hairs from bison #Y139 provide an isotopic history spanning across an annual cycle or encapsulating two dormant seasons.

## Discussion

Three conditions must be met for the forage maturation hypothesis to apply to patch selection by grazing herbivores: (1) short‐term rates of grazing intake must increase with plant biomass, (2) *ad libitum* limits on intake must be linked to indices of forage quality such as digestible energy or protein content, and (3) digestible energy or protein content must decline with increasing plant biomass within patches (Wilmshurst et al. 1995). By parameterizing a net protein intake model with bison functional response parameters and digestible protein values, we have shown that these conditions exist for bison and have demonstrated that bison select patches of low to intermediate biomass in a fire‐prone grassland.

Foraging areas within burned watersheds contained lower grass biomass than unburned watersheds with ~690 kg/ha, close to the model prediction. This result is also consistent with results reported by Wilmshurst and Fryxell (1995) and Langvatn and Hanley (1993) for elk and red deer (*Cervus elaphus*) in foraging trials. They conclude in both studies that patch selectivity optimized the rate of both digestible energy and protein intake. Additionally, Wilmshurst and Fryxell (1995) showed net digestible energy and protein intake strongly covaried, thus confirming that either of these chemical properties could be used in tests of the FMH. By incorporating a digestive constraint in our model, as suggested by Wilmshurst et al. (1995), we consider the implications of gut fill over an entire foraging period (10.7 h) on net protein intake in an experimental landscape with burned and unburned grassland watersheds.

Our optimality model suggests that bison should have spent all of their time in the most protein‐laden areas to maximize net protein intake in a day, which in our study area is burned tallgrass prairie. A concomitant movement study at KPBS with GPS‐collared adult female bison (Raynor 2015) corroborates this prediction because animal locations were primarily restricted to burned watersheds in the growing season. According to model predictions for net protein intake, bison realized markedly higher protein gain by grazing in burned watersheds over the growing season. In North American tallgrass prairie, land managers routinely conduct prescribed spring burning to remove senesced forage from previous years and provide lush, green forage for cattle. This land management protocol increases uniformity of spatial utilization in these pastures and may explain the substantial improvements in animal performance (Owensby and Smith 1979; Svejcar 1989).

Whether increased availability of green foliar tissue or high foliar nutrients due to burning are the primary factors responsible for restricted space use of grazers is a long‐unanswered question in studies of fire–grazer interactions (Woolfolk et al. 1975; Hobbs and Spowart 1984; Hobbs et al. 1991; Archibald 2008; Cromsigt and Olff 2008; Allred et al. 2011). Here, we used a model to predict where nutrient intake could be optimized and field measurements to understand patch selection. Our patch selection results indicate patches of low to intermediate biomass are selected over patches with greater biomass during the entire growing season (May to September). Used patches contained lower grass biomass and higher foliar protein than nearby, available patches. This result suggests structural and chemical properties of forage are not mutually exclusive in explaining grazer patch use in response to fire‐induced changes in forage characteristics. We posit that the initial pulse in green foliar accessibility and foliar nutrients instigated by fire sets the stage for restricted space use of grazers in fire‐managed mesic grasslands for the rest of the growing season. Moreover, after the postfire pulse, recursive grazing maintains available forage in a young and nutritious state for the remainder of the growing season, as predicted by the FMH.

Our study investigated the use of the FMH in predicting optimal foraging conditions for nutrient acquisition over three seasonal periods. Although patch selection was motivated by sites of low to intermediate biomass, the difference of grass biomass content between used and available sites diminished as the growing season advanced. Forb cover increased in foraging areas as grazing lawn senescence, suggesting that denser swards of ungrazed plants substituted the preferred foraging currency of low to intermediate biomass. Our interpretation of this behavioral adjustment is that avoidance of mature graminoid forage by large herbivores can last only as long as regrowth is possible.

Seasonal fluctuations in diets of vertebrate herbivores can arise from variation in temporal and spatial resource quality and abundance (Shipley et al. 2009). At KPBS, the most infrequently burned watersheds support a high proportion of cool‐season C_3_ grasses (Gibson and Hulbert 1987; Vinton et al. 1993). During the dormant season, observational studies and distribution of GPS‐collared bison show bison prefer watersheds that are not burned in the previous spring (Vinton et al. 1993; Raynor 2015; Raynor et al. 2015). Our stable isotope record of *δ* ^13^C using tail hairs of GPS‐collared female bison indicates their attraction to not‐burned watersheds in the dormant season is driven by the availability of cool‐season C_3_ grasses, consistent with an observation noted soon after bison introduction to KPBS (Vinton et al. 1993). Cool‐season C_3_ graminoids tend to occur in not‐burned tallgrass prairie with high vegetation structure (Coppedge and Shaw 1998), which likely decreases the accessibility of these forage resources and reduces foraging efficiency. During this period, we surmise that bison were compensating for low protein availability in forage by consuming higher amounts of low‐quality forage although this strategy may reduce short‐term nutrient intake as observed in other studies (Laca et al. 1994, 2001). However, the taxonomic resolution of our isotopic analysis did not separate C_3_ graminoids from nongrass C_3_ plants (e.g., trees, shrubs, and forbs) in the diet of our study animals. When preferred forage resources are scarce, grazing ruminants may supplement grass diets with forbs to maintain protein‐energy balance (Odadi et al. 2013). Regardless, the isotopic data from tail hair clearly show seasonal shifts in diet, from an exclusive C_4_ grass diet during the growing season, to a mixed C_4_/C_3_ diet in the dormant season that includes more C_3_ plants. This shift in foraging behavior at the end of the growing season also indicates that use of the behavioral preference for grass patches of low to intermediate biomass, the FMH, for predicting large grazer distribution may not apply to the dormant season.

We caution against the indiscriminant use of specific predictions of our optimality model for bison in tallgrass prairie for ungulates of sizes differing from those of the adult bison examined here. Underlying parameters of the functional response (bite size and cropping rate) vary allometrically among grazers (Gross et al. 1993; Wilmshurst et al. 2000) as well as activity budgets (Belovsky and Slade 1986), which could change the constraint curves substantially. Further, nutritional value and processing time can vary considerably among various food items available to herbivores (Whelan and Brown 2005), which could influence patterns of patch use.

### Implications for large herbivores in temperate environments

Plant phenology and environmental conditions strongly influence the physiological balance and foraging behavior of herbivores living in seasonal environments (Shrader et al. 2006; Parker et al. 2009). Unlike tropical grassland systems, where plant regrowth after grazing can occur with sufficient rainfall throughout the year (Fryxell 1991), availability of high‐quality forage is highly seasonal in temperate grasslands. At KPBS, bison selected feeding sites of low vegetation structural complexity during the growing season when regrowth was possible and then adjusted patch use behavior to patches of more complex vegetation structure in the dormant season where more effort is likely expended to reach nutrient requirements. The understanding of forage selection by herbivores in relation to nutritional constraints in burned and not‐burned grassland is an important step to predicting space use in temperate systems with an intact fire–grazer interaction. The determination of these constraints in grassland managed with fire may be critical for conservation programs by enabling us to model herbivore population dynamics effectively and to maintain the services provided in grassland ecosystems.

## Data Accessibility

Data will be deposited in the Konza Prairie Long‐term Ecological Research database: Konza Prairie LTER. Available online: https://portal.lternet.edu/nis/mapbrowse?packageid=knb-lter-knz.400.1. Data have been archived and are publicly accessible (Raynor et al. 2016).

## Conflict of Interest

None declared.

## Supporting information


**Appendix S1.** Tail hair methodology.
**Table S1.** Simple linear regression equations for relationship between digestible crude protein (%) and increasing grass biomass (g m^2^) in each burn status and season.
**Table S2.** Candidate models and parameter estimates for feeding‐site selection of bison at Konza Prairie, Manhattan, Kansas in spring 2012–2013, where selection for feeding sites were compared with random sites located in a random direction 50 m away.
**Table S3.** Candidate models and parameter estimates for feeding‐site selection of bison at Konza Prairie, Manhattan, Kansas in summer 2012–2013, where selection for feeding sites were compared with random sites located in a random direction 50 m away.
**Table S4.** Candidate models and parameter estimates for feeding‐site selection of bison at Konza Prairie, Manhattan, Kansas in fall 2012‐2013, where selection for feeding sites were compared with random sites located in a random direction 50 m away.
