# Supplementary material for: Foraging decisions underlying restricted space use: effects of fire and forage maturation on large herbivore nutrient uptake
Source: Ecol Evol. 2016 Jul 23;6(16):5843–53. doi: 10.1002/ece3.2304 (PMC4983596; doi:10.1002/ece3.2304)
Supplement: Supplementary file 1 — Appendix S1. Tail hair methodology. Table S1. Simple linear regression equations for relationship between digestible crude protein (%) and increasing grass biomass (g m2) in each burn status and season. Table S2. Candidate models and parameter estimates for feeding‐site selection of bison at Konza Prairie, Manhattan, Kansas in spring 2012–2013, where selection for feeding sites were compared with random sites located in a random direction 50 m away. Table S3. Candidate models and parameter estimates for feeding‐site selection of bison at Konza Prairie, Manhattan, Kansas in summer 2012–2013, where selection for feeding sites were compared with random sites located in a random direction 50 m away. Table S4. Candidate models and parameter estimates for feeding‐site selection of bison at Konza Prairie, Manhattan, Kansas in fall 2012‐2013, where selection for feeding sites were compared with random sites located in a random direction 50 m away. [file ECE3-6-5843-s001.docx]

***Appendix***

*Tail hair methodology*

Individual 5-mm hair sections were measured for the stable carbon isotope ratio (δ^13^C) at the Stable Isotope Mass Spectrometry Laboratory (SIMSL) at Kansas State University. Samples were combusted with a CE1110 elemental analyzer (Carlo Erba Instruments, Milan, Italy) and coupled to a Delta Plus mass spectrometer (Thermo Electron Corporation, Bremen, Germany) for isotope analysis using a ConFlo II Universal Interface (Thermo Electron Corporation, Bremen, Germany). The isotopic ratio of samples was calculated using delta notation as:

$\delta=[(\frac{Rsample}{Rstandard}$ -1) * 1000] , (Equation 2)

where *R* is the ratio of the heavy to light isotope for the sample and standard (PDB), respectively. The within-run variability estimated as the standard deviation of working standards was always ≤ 0·05%, and the between run variability, estimated as the difference between the measured value of a working standard and its calibrated value, was always 0·05%.

Strongly delineated isotopic signals occur in the δ^13^C ratios of plants, reflecting differences associated with C_3_ and C_4_ photosynthetic pathways. Most C_4_ plants have δ^13^C values between -11 and -14‰, whereas most C_3_ plants have δ^13^C values between -25 and -29‰ (Dawson *et al.* 2002). We classify diets of primarily C_4_ plant as those represented by hair follicle δ^13^C values greater than -14‰, while values smaller than -14‰ represented mixed C_4_-C_3_ plant diets unless a value of -25‰ was reached. Linear and polynomial regression was performed to assess the δ^13^C distribution across the tail hair length. Non-linear changes in δ^13^C signatures throughout a year indicate a diet shift from plants. Log-likelihood estimates were compared for the linear or polynomial distributions for each tail hair. The distribution with the lowest AICc value was retained for interpretation. Annual average tail hair length in our samples was 11·72 ± 0·16 cm. The hair growth rate for cattle is ~0·69-1·06 mm d^-1^ (Schwertl, Auerswald & Schnyder 2003); we presume similar or somewhat slower tail hair growth rates for bison given generally lower quality food, and that our data encapsulate isotopic history spanning from late winter through summer for all four years in each animal.

Table S1. Simple linear regression equations for relationship between digestible crude protein (%) and increasing grass biomass (g m^2^) in each burn status and season. Data was pooled from bison foraging sites with burned and not-burned watersheds at Konza Prairie Biological Station, Manhattan, Kansas, U.S.A.

| Season | Burn Status | Equation | DF | F | P | R^2^ |
| --- | --- | --- | --- | --- | --- | --- |
| Growing | Yes | y=6.66-0.01x | 1,65 | 15.10 | <0.0001 | 0.19 |
| Growing | No | y=5.28-0.01x | 1,8 | 7.12 | 0.03 | 0.40 |
| Peak Growth | Yes | y=5.04-0.01x | 1,83 | 10.47 | 0.002 | 0.10 |
| Peak Growth | No | y=4.68-0.01x | 1,37 | 19.28 | <0.0001 | 0.32 |
| Dormant | Yes | y=1.38-0.01x | 1,14 | 3.07 | 0.10 | 0.12 |
| Dormant | No | y=1.10-0.005x | 1,36 | 7.4 | 0.01 | 0.15 |

Table S2. Candidate models and parameter estimates for feeding-site selection of bison at Konza Prairie, Manhattan, Kansas in spring 2012-2013, where selection for feeding sites were compared with random sites located in a random direction 50 m away. Interaction term parameter estimates (95% CI) are indicate below table.

| Model |  | Forbs | Grass | Litter | Green | df | AICc | ΔAICc | ω*_i_* |  |
| --- | --- | --- | --- | --- | --- | --- | --- | --- | --- | --- |
| Forb + Grass |  | -38·10 (-69·21,-6·99) | -10·34 (-18·11,-2·57) |  |  | 2 | 19·12 | 0·00 | 0·88 |  |
| Forb + Grass + Litter + Green |  | -39·50 (-72.81, -6.18) | -10·44 (-18.51, -2.37) | 0.53 (-28.25, 29.31) | 2·99 (-10.29, 16.28) | 4 | 23.30 | 4·18 | 0·11 |  |
| Forb + |  | -24·26 (-41.12, -7.39) |  |  |  | 1 | 31·04 | 11·91 | 0·0 |  |
| Forb + Litter |  | -23·99 (-41.44, -6.54) |  | -11·75 (-32.12, 8.62) |  | 2 | 31·61 | 12·49 | 0·0 |  |
| Forb + Green |  | -24·61 (-41·79, -7·42) |  |  | 1·24 (-6·89, 9·37) | 2 | 33·05 | 13·93 | 0·00 |  |
| Forbs + Green + Forbs x Green* |  | 33.05 (-49.67, 115.77) |  |  | 8.18(-4.98, 21.35) | 3 | 33·31 | 14·18 | 0·00 |  |
| Grass + Green + Grass x Green# |  |  | -53.24 (-100.60, -5.87) |  | -33.01 (-63.41, -2.61) | 3 | 46·17 | 27·05 | 0·00 |  |
| Grass |  |  | -3·79 (-7·41, -0·16) |  |  | 1 | 52·43 | 33·31 | 0·00 |  |
| Grass + Green |  |  | -3·95 (-7·72, -0·17) |  | -3·07 (-9·70, 3·55) | 2 | 53·65 | 34·53 | 0·00 |  |
| Litter |  |  |  | -12·42 (-29·34, 4·49) |  | 1 | 54·81 | 35·69 | 0·00 |  |
| Litter + Green + Litter x Green |  |  |  | -116.89 (-256.41, 22.63) | -4.51 (-11.73, 2.72) | 3 | 54·84 | 35·71 | 0·00 |  |
| Litter + Green |  |  |  | -12·35 (-28·95, 4·25) | 2·77 (-8·93, 3·38) | 2 | 56·09 | 36·97 | 0·00 |  |
| Green |  |  |  |  | -2.55 (-8.34, 3.24) | 1 | 56·73 | 37·61 | 0·00 |  |
| Null (without covariates) |  |  |  |  |  | 0 | 103.36 | 86·94 | 0·00 |  |
|  |  |  |  |  |  |  |  |  |  |  |

*-76.82 (-191.59, 37.95), #60.85 (5.98, 115.73), **135.94 (-38.03, 309.91)

Table S3. Candidate models and parameter estimates for feeding-site selection of bison at Konza Prairie, Manhattan, Kansas in summer 2012-2013, where selection for feeding sites were compared with random sites located in a random direction 50 m away. Interaction term parameter estimates (95% CI) are indicate below table.

| Model | Forbs | Grass | Litter | Green | df | AICc | ΔAICc | ω*_i_* |
| --- | --- | --- | --- | --- | --- | --- | --- | --- |
| Forb + Grass | -21·39 (-30.90, -11.88) | -8·52 (-13.89, -3.14) |  |  | 2 | 50·29 | 0·00 | 0·58 |
| Forb + Grass + Litter + Green | -22·42 (-33.13, -11.73) | -8·54 (-14.04, -3.03) | -8.68 (-20.05, 2.69) | -1·28 (-5.21, 2.66) | 4 | 50·98 | 0·69 | 0·41 |
| Forb + Green + Forbs x Green* | -45·62 (-84·07, -7·18) |  |  | -10·61 (-19·86, -1·37) | 3 | 60·16 | 9·88 | 0·00 |
| Forb + Litter | -15·76 (-23.62, -7.91) |  | -9.42 (-19.86, 1.02) |  | 2 | 64·16 | 13·87 | 0·00 |
| Forb | -14·44 (-21.68, -7.21) |  |  |  | 1 | 65·64 | 15·35 | 0·00 |
| Forb + Green | -13·23 (-20.67, -5.79) |  |  | -1·97 (-5.49, 1.55) | 2 | 66·42 | 16·14 | 0·00 |
| Grass + Green + Grass x Green# |  | 4.99 (-5.38, 15.35) |  | 1.97 (-6.47, 10.41) | 3 | 81·44 | 31·15 | 0·00 |
| Grass + Green |  | -3.08 (-6.12, -0.05) |  | -4.66 (-7.95, -1.37) | 2 | 81·76 | 31·47 | 0·00 |
| Green |  |  |  | -4.20 (-7.29, -1.11) | 1 | 84·38 | 34·10 | 0·00 |
| Green + Litter |  |  | -4.81 (-13.22, 3.60) | -4·28 (-7.42, -1.14) | 2 | 85·07 | 34·79 | 0·00 |
| Litter + Green + Litter x Green** |  |  | 7.86 (-25.21, 40.93) | -3·66 (-7.04, -0.27) | 3 | 86·49 | 36·20 | 0·00 |
| Grass |  | -2.40 (-4.99, 0.19) |  |  | 1 | 89·88 | 39·59 | 0·00 |
| Grass + Litter |  | -2·28 (-4.89, 0.33) |  | -3.41 (-10.84, 4.02) | 2 | 91·09 | 40·81 | 0·00 |
| Litter |  |  | -4.19 (-11.62, 3.25) |  | 1 | 92·22 | 41·93 | 0·00 |
| Null (without covariates) |  |  |  |  | 0 | 177·65 | 127·37 | 0·00 |

*53.16 (-6.04, 112.35), #-13.83 (-31.54, 3.88), **-24.95 (-87.62, 37.72)

| Model | Forbs | Grass | Litter | Green | df | AICc | ΔAICc | ω*_i_* |
| --- | --- | --- | --- | --- | --- | --- | --- | --- |
| Litter |  |  | -2.34 (-9.07, 4.38) |  | 1 | 39·69 | 0·00 | 0·15 |
| Forbs | -2.44 (-9.51, 4.64) |  |  |  | 1 | 39·85 | 0·16 | 0·14 |
| Green |  |  |  | 2.25 (-4.97, 9.47) | 1 | 78·80 | 1·87 | 0·11 |
| Grass |  |  |  | 0·18 (-3.94, 4.30) | 1 | 79·00 | 2·05 | 0·10 |
| Forb + Litter | -3.30 (-10.74, 4.13) |  | -2.90 (-9.65, 3.86) |  | 2 | 79·10 | 2·15 | 0·09 |
| Forb + Green | -2·95 (-10.15, 4.25) |  |  | 2.85 (-4.63, 10.33) | 2 | 80·40 | 3·46 | 0·05 |
| Litter + Green |  |  | -2.41 (-9.54, 4.73) | 2.20 (-5.05, 9.46) | 2 | 80·60 | 3·62 | 0·04 |
| Grass + Litter |  | -0·77 (-5.56, 4.00) | -2·77 (-9.86, 4.32) |  | 2 | 80·70 | 3·78 | 0·04 |
| Forb + Grass | -2.75 (-10.49, 4.99) | -0.47 (-5.05, 4.11) |  |  | 2 | 80·70 | 3·79 | 0·04 |
| Grass + Green |  | 0.19 (-3.94, 4.34) |  | 2.25 (-4.97, 9.47) | 2 | 81·00 | 4·07 | 0·04 |
| Forb + Green + Forb x Green | 2.66 (-9.92, 15.24) |  |  | 8·69 (-4.73, 22.10) | 3 | 81·20 | 4·29 | 0·03 |
| Litter + Green + Litter x Green |  |  | -3·80 (-14.65, 7.04) | 0.08 (-8.47, 8.62) | 3 | 82·00 | 5·10 | 0·02 |
| Forb + Grass + Litter + Green | -7·42 (-17.47, 2.63) | -3.74 (-10.26, 2.78) | -5·71 (-13.86, 2.43) | 3·35 (-4.31, 11.01) | 3 | 82·30 | 5·34 | 0·02 |
| Grass + Green + Grass x Green |  | 0·98 (-4.51, 6.47) |  | 7.19 (-16.33, 30.71) | 3 | 82·70 | 5·76 | 0·02 |
| Null (without covariates) |  |  |  |  | 0 | 83·00 | 6·06 | 0·01 |

Table S4. Candidate models and parameter estimates for feeding-site selection of bison at Konza Prairie, Manhattan, Kansas in fall 2012-2013, where selection for feeding sites were compared with random sites located in a random direction 50 m away. Interaction term parameter estimates (95% CI) are indicated in the table below.

*-23.79 (-69.14, 21.55), #-8.73 (-47.92, 30.46), **40.18 (-51.57, 131.94)

Literature Cited

Dawson, T.E., Mambelli, S., Plamboeck, A.H., Templer, P.H. & Tu, K.P. (2002) Stable isotopes in plant ecology. *Annual Review of Ecology and Systematics***,** 507-559.

Schwertl, M., Auerswald, K. & Schnyder, H. (2003) Reconstruction of the isotopic history of animal diets by hair segmental analysis. *Rapid Communications in Mass Spectrometry,* **17,** 1312-1318.
